# Neonatal cerebral infarction: A case report

**DOI:** 10.1097/MD.0000000000043759

**Published:** 2025-08-01

**Authors:** Zhonghua Hu, Chengchao Fang, Yueyan Mao

**Affiliations:** a Department of Pediatrics, The First Affiliated Hospital of Linping District , Hangzhou, China.

**Keywords:** case, infant, neonatal, neonatal cerebral infarction

## Abstract

**Rationale:**

Neonatal cerebral infarction can be focal or widespread, clinically silent or neurologically catastrophic, embolic or thrombotic, prenatalor postnatal, and hemorrhagic or occlusive.

**Patient concerns:**

A neonatal was diagnosed with neonatal cerebral infarction after birth.

**Diagnoses:**

A large area of cerebral infarction was found through brain computed tomography and cranial magnetic resonance imaging examinations.

**Interventions:**

The child is restricted from fluids, and dehydration reduces intracranial pressure.

**Outcomes:**

Emiparesis may be absent in the neonatal period. However, over the next several months, an asymmetry of left hemiplegia may gradually become evident.

**Lessons:**

At present we cannot accurately predict which infants with neonatal stroke will have significantly abnormal language development. Therefore, the most important thing for the patient is to have regular follow-up visits at the outpatient clinic, and if necessary, to engage in timely rehabilitation exercise training.

## 1. Introduction

Neonatal cerebral infarction (NCI), also known as neonatal stroke, typically refers to the occurrence of 1 or more branches of the cerebral blood vessels in newborns, from 28 weeks of gestation to 28 days after birth, becoming occluded for various reasons, leading to ischemic damage in the corresponding blood supply areas of the brain tissue.^[1]^ It is classified into ischemic and hemorrhagic cerebral infarction. Clinically, it is not uncommon. Due to the lack of specific clinical symptoms at birth, NCI often occurs in seemingly healthy full-term newborns, with early symptoms being mild or absent, and motor or cognitive function impairments may only appear months after birth.^[2]^ Therefore, early diagnosis is quite challenging. Although infants with neonatal stroke can survive, it can lead to cerebral palsy, delayed brain development, and residual motor or cognitive function impairments, severely affecting the quality of life of the affected children. This case aims to highlight diagnostic challenges of NCI with initially normal Apgar scores. Thus, early diagnosis and timely, appropriate treatment are key to alleviating and reducing sequelae.

## 2. Case presentation

Written informed consent was obtained from both parents, and this study was approved by the Ethics Committee of Zhejiang University Second Affiliated Hospital Linping Campus (Approval number: 2024056). Neonatal intensive care unit (NICU) admission was protocolized for neonates with: metabolic acidosis (pH < 7.30 + lactate > 5 mmol/L), seizures, or meconium aspiration risk. This case met all 3 criteria. Initial blood gas was obtained from umbilical artery cord blood within 5 minutes of delivery. The patient is a male infant, 33 minutes old, admitted 33 minutes after birth due to grade III meconium-stained amniotic fluid (MSAF). Although the infant had normal Apgar scores, the decision to admit to the NICU was based on the following high-risk factors:metabolic acidosis: Initial umbilical artery pH was 7.26 and lactate was 5.5 mmol/L, meeting our institutional NICU admission criteria for metabolic disturbances. As per American Academy of Pediatrics recommendations, neonates with MSAF III require at least 24 to 48 hours of observation, especially when accompanied by acidosis or abnormal fetal monitoring. The infant is the fifth child from the third pregnancy, delivered by cesarean section at 37 weeks and 1 day due to “fetal distress and a scarred uterus.” Birth weight was 3060 g, with signs of intrauterine distress, no premature rupture of membranes, and moderate MSAF. Apgar scores were 10 at 1 minute and 10 at 5 minutes, with no history of asphyxia or resuscitation. After birth, the infant’s response was acceptable, with a weak cry, the infant did not exhibit classic meconium aspiration syndrome symptoms (e.g., cyanosis, retractions), no fever or temperature elevation, no signs of respiratory distress, moaning, or cyanosis, no agitation or irritability, no seizures or excessive crying, and no vomiting or abdominal distension. For further diagnosis and treatment, the infant is planned to be admitted as a “high-risk infant.” After hospitalization, blood gas analysis indicated a pH of 7.26, carbon dioxide partial pressure of 27.2 mm Hg, oxygen partial pressure of 94 mm Hg, hemoglobin of 15 g/dL, sodium ion of 141 mmol/L, potassium ion of 3.2 mmol/L, lactate of 5.5 mmol/L, and actual bicarbonate deficit of −13. The complete blood count showed white blood cells at 15.8 × 10^9^/L, C-reactive protein <0.2, and no abnormalities in cytomegalovirus and Toxoplasma gondii Others Rubella virus Cytomegalovirus Herpes simplex virus tests. Serial glucose monitoring (pre-feed values: 3.2–4.1 mmol/L) and cortisol levels (AM: 280 nmol/L, PM: 150 nmol/L) remained within normal ranges, excluding hypoglycemia or adrenal insufficiency. Blood biochemistry showed no significant abnormalities, coagulation tests were normal, d-dimer was 1.09 mg/L, and routine stool and urine tests showed no abnormalities. Sodium bicarbonate was administered to correct acidosis, and a follow-up blood gas analysis showed a pH of 7.49, carbon dioxide partial pressure of 22.7 mm Hg, oxygen partial pressure of 69 mm Hg, hemoglobin of 15 g/dL, sodium ion of 135 mmol/L, potassium ion of 4.5 mmol/L, lactate of 6.8 mmol/L, and actual bicarbonate deficit of −3, blood culture is negative, 30-minute video-electroencephalogram (EEG) revealed left hemispheric slowing (5–6 Hz) without epileptiform discharges, all thrombophilia screening results were within normal neonatal reference ranges, no pathological thrombophilic risk factors were identified. He mother was G5P3 with 2 prior term deliveries (healthy daughters), 2 first-trimester induced abortions, and no fetal/neonatal losses. Family history was negative for inherited thrombotic or neurodevelopmental disorders.

## 3. Treatment

**Figure 1.**
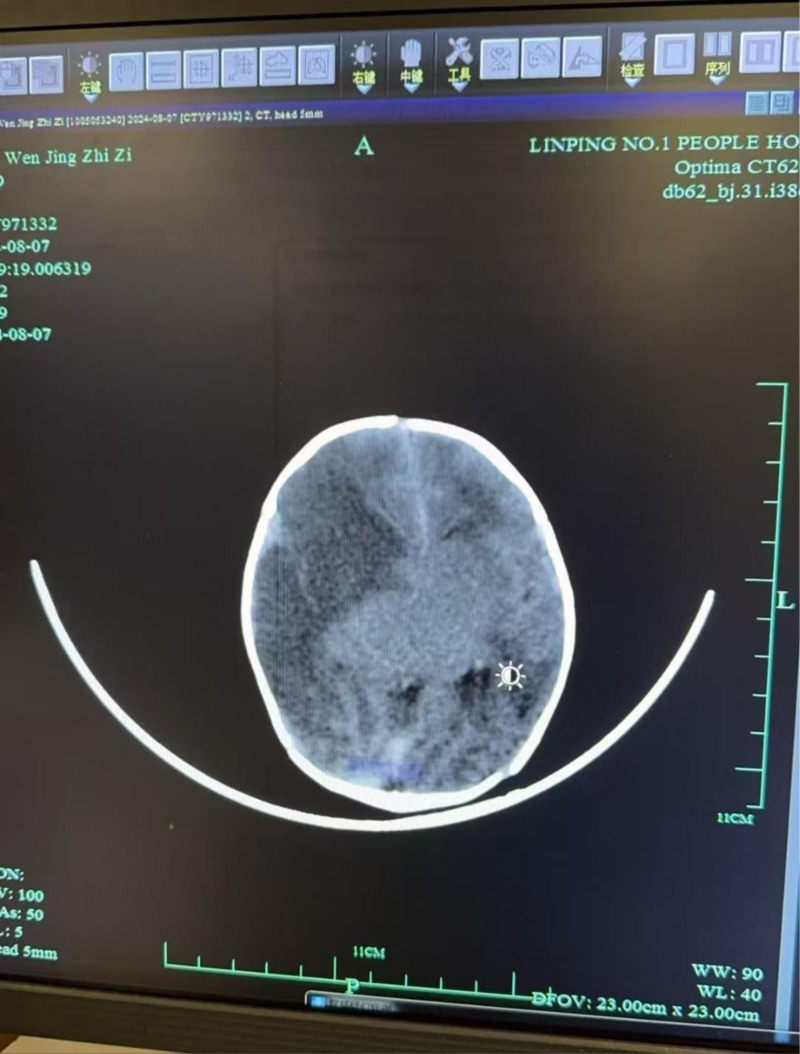
The CT of neonatal cerebral infarction. CT = computed tomography.

After admission, it was found that the child’s crying was not very melodious, and there were occasional seizures lasting about 30 seconds. The muscle tone was slightly elevated, and the child was easily irritable. Considering that the child had no history of birth asphyxia, grade III MSAF and blood gas analysis indicated metabolic acidosis, and the child exhibited neurological symptoms, a comprehensive emergency computed tomography scan of the brain was performed to rule out intracranial hemorrhage. The results revealed a large area of cerebral infarction, as shown in Figure 1. Therefore, we improved the magnetic resonance examination to confirm our previous results, but the child’s EEG showed no significant abnormalities. Due to the child’s neurological symptoms, midazolam was administered for sedation, dehydration, and to reduce intracranial pressure. The child did not experience seizures and was discharged for follow-up. Later, during outpatient follow-up, it was found that the child had poor movement in the contralateral limbs, and outpatient rehabilitation training was provided. Three months: EEG showed no epileptiform discharges. Six months: Left-sided hypertonia detected on motor assessment (Gesell scale scores included).

Eighteen months: Expressive language delay (e.g., <5 words) documented. Imaging-clinical correlation: Figure 1 (computed tomography): Now annotated to highlight a hypodense lesion in the right middle cerebral artery territory, consistent with the infant’s left hemiparesis.

## 4. Discussion

NCI includes perinatal arterial ischemic infarction, cerebral venous sinus thrombosis, and hemorrhagic infarction. The etiology of NCI is complex, and the risk factors and prognostic factors remain unclear.^[3]^ It may be due to a combination of various high-risk factors. Studies have shown that the probability of NCI occurring after cesarean delivery is 3 to 4 times higher than that after natural delivery, and cesarean surgery itself is a high-risk factor for thrombosis, indicating that cesarean delivery is a risk factor for cerebral infarction. In this group of cases, cesarean delivery was performed due to factors such as fetal distress and maternal scar uterus, which may have been caused by placental vascular abnormalities or coagulation disorders.^[3,4]^ Additionally, the hypercoagulable state of fetal blood, placental inflammation, and reduced placental blood flow may be related to NCI. The clinical manifestations of NCI are atypical, with nonspecific neurological symptoms and signs (such as respiratory failure, temperature instability) as well as typical neurological symptoms (seizures, abnormal postures), all of which may indicate an interruption in the supply or demand of oxygen to the brain of the newborn.^[5,6]^ Previous studies have shown that seizures are a common clinical manifestation, with multifocal clonic seizures being the main form of attack, and they are significant for localization diagnosis, with left hemisphere infarction being more common. In this group of cases, the initial symptom in affected infants was seizures, consistent with previous studies.^[7]^ Left hemisphere infarcts in neonates often manifest as delayed right hemiparesis and language deficits (e.g., this case’s speech delay at 12 months). EEG’s prognostic value remains debated, but absence of seizures predicts better outcomes. The EEG results of NCI may be normal or show sharp waves, focal slowing, or periodic unilateral epileptiform discharges. In this group of cases, surviving infants monitored with EEG at 3 months and 6 months did not show any epileptiform discharges. This may be related to our small sample size, and further follow-up observation is needed.

## 5. Conclusion

In summary, regular prenatal follow-up and examinations, active prevention of infections during pregnancy, control of gestational diabetes and hypertension, and vigilance for neonatal ABO hemolysis should be emphasized. When specific or nonspecific neurological symptoms appear in newborns postpartum, EEG, head ultrasound, and other examinations should be conducted as soon as possible. If cerebral infarction is suspected, head magnetic resonance imaging (including diffusion weighted imaging and magnetic resonance angiography) should be completed promptly to facilitate early diagnosis and treatment, early intervention, and improved prognosis.

## 6. Limitation

While this detailed case analysis provides important clinical insights, we acknowledge the need for larger prospective studies. In the future, large-sample and multicenter studies can be conducted.

## Author contributions

**Conceptualization:** Chengchao Fang.

**Data curation:** Chengchao Fang.

**Methodology:** Chengchao Fang.

**Resources:** Yueyan Mao.

**Software:** Yueyan Mao.

**Supervision:** Yueyan Mao.

**Validation:** Yueyan Mao.

**Writing – original draft:** Zhonghua Hu.
